# Association of Serum Osteoprotegerin Level With Myocardial Injury and Cardiovascular Calcification in Chronic Kidney Disease Patients

**DOI:** 10.3389/fmed.2022.814970

**Published:** 2022-06-22

**Authors:** Kamal M. Okasha, Mohamed Hussein Aboufreikha, Waleed Elrefaey, Medhat M. Ashmawy, Heba Mourad, Mohamed A. Elsebaey, Mohammed H. Elnaggar, Raghda Gabr Mashaal, Sama Metwally, Shaimaa Samir Amin Mashal, Neveen A. Shalaby, Shireen Ali Elhoseny, Amr Alkassas, Mohammed Elbarbary, Osama Shoeib, Dina A. Ali, Nivin Baiomy, Sherein M. Alnabawy

**Affiliations:** ^1^Internal Medicine Department, Faculty of Medicine, Tanta University, Tanta, Egypt; ^2^Cardiovascular Medicine Department, Faculty of Medicine, Tanta University, Tanta, Egypt; ^3^Clinical Pathology Department, Faculty of Medicine, Tanta University, Tanta, Egypt

**Keywords:** osteoprotegerin, troponin T, cardiovascular calcifications, myocardial injury, chronic kidney disease

## Abstract

**Background:**

Chronic kidney disease has emerged as a significant independent risk factor for cardiovascular disease. Cardiovascular calcification is an active process involving a complex interaction of inducers and inhibitors. High sensitivity cardiac troponin T assay detects troponin T with higher sensitivity and precision at an earlier point of time than the conventional assays, and is associated with poor outcomes. Serum osteoprotegerin is classed as an inhibitory factor for cardiovascular calcification. It is involved in the pathological processes of vascular damage and linked to the excess cardiovascular morbidity. The aim of the present study was to evaluate the extent of cardiovascular calcification and serum high sensitivity cardiac troponin T level, and their association with serum osteoprotegerin level in patients with chronic kidney disease stages 3–5.

**Methods:**

90 chronic kidney disease patients were enrolled in this study, and they were divided into two groups: group (1) included 45 non-dialysis-dependent chronic kidney disease patients (stages 3–5) and group (2) included 45 chronic hemodialysis patients. Each group further subdivided according to the presence of cardiovascular calcification into subgroup A and B. Vascular calcifications were assessed by lateral lumbar, pelvis and hands X-ray radiographs. Valvular calcification was assessed by echocardiography. Serum cardiac troponin T was measured by high sensitivity assay and serum osteoprotegerin was measured by ELISA.

**Results:**

Cardiovascular calcification distribution was 22.2% in group (1) and 33.3% in group (2). Serum osteoprotegerin and troponin T in calcification groups (1A and 2A) were significantly higher than non-calcification groups (1B and 2B; *P* < 0.001). Osteoprotegerin correlated positively with high sensitivity cardiac troponin T (rs = 0.72, *P* < 0.001). cardiovascular calcification correlated positively with osteoprotegerin, troponin T, and phosphorus. osteoprotegerin and phosphorus were significant independent predictors of cardiovascular calcification at cut-off values ≥4.6 ng/L and ≥6.95 mg/dl, respectively (*P* < 0.001). Serum phosphorus and creatinine were independent predictors of osteoprotegerin (*P* < 0.001 and 0.048, respectively).

**Conclusion:**

Osteoprotegerin is strongly associated with cardiovascular calcification and high sensitivity cardiac troponin T. In addition, there is a positive association between calcification and troponin T. This suggests a role for osteoprotegerin in the pathogenesis and risk stratification of cardiovascular calcification and myocardial injury in chronic kidney disease patients with a potential role as a therapeutic target.

## Introduction

Cardiovascular disease (CVD) is considered a leading cause of morbidity and mortality in chronic kidney disease (CKD) patients, and the incidence of cardiac mortality increases with decline in renal function (1). The risk of developing CVD in patients with CKD exceeds the risk of reaching end-stage kidney disease (ESKD) (2), as the majority of these patients will ultimately die from a cardiovascular event prior to progression to ESKD (3).

This increased incidence is due to the extended exposure of CKD patients to traditional as well as non-traditional risk factors (4). Traditional cardiovascular risk factors include age, diabetes mellitus, hypertension, smoking, and dyslipidemia (5), and non-traditional risk factors include oxidative stress, inflammation, progressive atherosclerosis and bone and mineral metabolism derangement (6).

Cardiovascular death results from heart failure and fatal arrhythmias, in addition to fatal atherosclerosis-related complications such as myocardial infarction and stroke especially in advanced CKD stages (7). Cardiovascular calcification (CVC) is increasing with progressive decline in kidney function and sharing in the increased cardiovascular risk (8). Vascular and valvular calcifications in CKD occur partly due to disturbed mineral metabolism with passive deposition of calcium and the therapies used to control it (9), and also due to an active process of osteogenesis in vascular smooth muscle cells (10).

Cardiac troponin T (cTnT) is considered a sensitive and specific biomarker of myocardial injury and an index of irreversible myocardial change (11). High sensitivity cardiac troponin T (hs-cTnT) assay may be useful for studying the earliest stages of heart disease in CKD patients, as it detects much lower levels of myocardial injury (up to 10 fold lower levels) than previous assays (12).

A rise in troponins compared with previous persistent elevated values, with serial measurements, has been considered to help differentiate acute myocardial injury from chronic elevations of cTnT in patients with advanced CKD (13). In addition, hs-cTnT has a strong association with changes of left ventricular structure and diastolic dysfunction (14).

Osteoprotegerin (OPG) is a soluble member belonging to the tumor necrosis factor (TNF) receptor superfamily, and it has pleiotropic effects on bone metabolism, immune system and endocrine function (15). It is a calcification natural inhibitor which prevents osteoclastic bone resorption by binding to the receptor activator of nuclear factor-κB ligand (RANKL), working as a decoy receptor leading to competitive inhibition of RANKL interaction with its receptor (16).

Adverse cardiovascular outcomes in CKD were proved to be associated with higher OPG levels (17). A crosstalk between the bone and the vasculature exists, its pathological expression is the close association between bone turnover, vascular calcification and cardiovascular events in CKD patients (18).

To determine the relationship between serum OPG, as a marker of bone-mineral metabolism, and the extent of cardiovascular calcification and hs-cTnT level as a myocardial injury marker in CKD patients, this study was carried out.

## Methods

All participants were enrolled in this observational cross-sectional study, which was conducted at Internal Medicine Department (Nephrology Unit). The patients were recruited from nephrology inpatient wards, outpatient clinic and hemodialysis unit within a period of 1 year from January 2017 to January 2018.

### Study Population

This study included 90 chronic kidney disease patients, who were divided into two groups: group (1) included 45 non-dialysis-dependent CKD patients (CKD ND) stages 3–5, and group (2) included 45 patients with CKD on maintenance hemodialysis (CKD 5HD). Each group was subdivided according to the presence of CVC into: a subgroup A (patients with CVC) and a subgroup B (patients without CVC).

#### Inclusion and Exclusion Criteria

- Non-dialysis CKD patients with estimated glomerular filtration rate (eGFR) <60 ml/min/1.73 m^2^ and chronic hemodialysis patients (dialysis duration ≥6 months) were included in the study for group 1 and group 2, respectively.
- We excluded the following:

patients <18 years old, pregnant females, CKD patients with eGFR ≥60 ml/min/1.73 m^2^ or with hemodialysis period <6 months, patients with heart failure [left ventricular ejection fraction (EF) <35%], patients with symptoms of acute coronary syndrome, acute coronary event or any cardiac procedure within 1 month before enrollment in the study, recent infection within 1 month before enrollment in the study, patients using corticosteroids or immunosuppressive agents and patients who have malignant tumors.

Patients included in the study were subjected to: thorough history taking and complete clinical examination, emphasizing on: etiology and duration of CKD, history of cardiovascular disease, smoking history, drugs that were taken at entry to the study and duration and prescription of dialysis therapy; all group 2 patients were dialyzed for ≥6 months *via* arteriovenous fistula, three times/week for 4 h session using polysulfone low flux dialyzer 1.6 m^2^ surface area, with blood flow 300–350 ml/min and dialysate flow 500 ml/min and dialysate calcium concentration 1.5 mmol/L, using heparin as anticoagulant with tailored doses according to each case and bicarbonate based dialysate.

Laboratory investigations, included complete blood count, serum creatinine, blood urea, eGFR [using Modification of Diet in Renal Disease (MDRD) equation] (19), serum albumin, C-reactive protein (CRP), serum total cholesterol and triglycerides, serum total alkaline phosphatase (ALP), intact parathyroid hormone (iPTH), serum corrected total calcium, phosphorus and magnesium.

Assessment of hemodialysis adequacy was done using single pool Kt/V by the second generation Daugirdas formula (for hemodialysis patients group) (20). Electrocardiography (ECG) was done to exclude any abnormal new findings concerning acute myocardial ischemia, infarction or heart failure.

Serum OPG level was measured using a double-antibody sandwich enzyme-linked immunosorbent assay (ELISA). SunRed biological technology Catalog No. 201-12-1559. Sensitivity: 2.044 ng/L. Assay range 2.5–720 ng/L. Intra-assay CV <10%, Inter- assay CV <12% (21). Serum cardiac troponin T was measured using high sensitivity immunoassay on TOSOH AIA-1800ST (22).

### Radiological Techniques

#### Echocardiography

A two-dimensional transthoracic echocardiographic study was performed on each patient. Digital images were acquired in the long-axis and short-axis parasternal views and the apical four and two chamber views. The presence of aortic and mitral valve calcification, separately, was determined visually by a single experienced cardiologist and assessed as being present or absent. Valvular calcification was defined as bright echoes of more than 1 mm on 1 or more cusps of the aortic and the mitral valves.

#### Lateral Lumbar Plain X-Ray Radiography

Lateral lumbar radiography was performed in the standing position using standard radiographic equipment to assess abdominal aorta calcifications. Calcification of the aorta was graded using a previously validated system (Kauppila score) in which both the location and the severity of calcific deposits at each lumbar vertebral segment (L1–L4) were evaluated.

For this validated 24-point abdominal aortic calcification score (AACS), calcified deposits along the anterior and posterior longitudinal walls of the abdominal aorta adjacent to each lumbar vertebra from L1 to L4 were assessed using the midpoint of the intervertebral space above and below the vertebrae as the boundaries (23).

### Grading of Calcifications Was as Follows

0 for no Aortic Calcific Deposits; 1 for Small Scattered Calcific Deposits less than One-third of the Corresponding Length of the Vertebral Level; 2 for Medium Quantity of Calcific Deposits About One-third or More, but less than two thirds of the Corresponding Vertebral Length; 3 for Severe Quantity of Calcifications of more than Two-thirds or More of the Corresponding Vertebral Lengths. The Scores, Obtained Separately for the Anterior and Posterior Walls, Resulted in a Range From 0 to 6 for Each Vertebral Level and 0–24 for the Total Score (24).

#### Anteroposterior Pelvis and Hands Plain X-Ray Radiography

A simple vascular calcification score was used to assess peripheral vascular calcifications in the pelvis and both hand. This vascular calcification score (Adragao score) was evaluated in plain radiographic films of pelvis and hands.

The pelvis radiographic films evaluated iliac and femoral arteries and were divided into four sections by two imaginary lines: a horizontal line over the upper limit of both femoral heads and a median vertical line over the vertebral column. The films of the hands evaluated radial and digital arteries and were divided, for each hand, by a horizontal line over the upper limit of the metacarpal bones. The presence of linear calcifications in each section was counted as 1 and its absence as 0. The final score was the sum of all the sections, ranging from 0 to 8 (25).

### Disclaimer

- An approval was obtained from Research Ethics Committee of Faculty of Medicine Tanta University to conduct this study and to use the facilities in the hospitals. Reference number is 2443/03/14.
- Informed written consents were obtained from all patients after full explanation of the benefits and risks of the study. Privacy of all patients' data was granted by a special code number for every patient's file that included all investigations.

### Statistical Analysis

The collected data were organized, tabulated, and statistically analyzed using SPSS software version 20.

– For quantitative data, the Shapiro-Wilk test for normality was performed.
– For data that were not normally distributed median and interquartile range (expressed as 25th−75th percentiles) were calculated and Mann-Whitney and Kruskall-Wallis tests were used.
– For normally distributed data, values were expressed as mean ± standard deviation and Independent samples *T*-test and one way ANOVA were performed for comparison between groups.
– For qualitative data, Pearson's Chi square test was used to examine association between two variables.
– Spearman's rank correlation was done to test associations of the studied variables with osteoprotegerin and cardiovascular calcifications.
– Binary logistic regression and receiver operation characteristic (ROC) curve analysis were carried out to test the power and validity of some studied variables to predict cardiovascular calcification.
– In addition, multivariate regression analysis was done to predict OPG level.
– Significance was adopted at *P* < 0.05 for interpretation of results of the tests.

## Results

This observational cross-sectional study included 90 patients, who were divided into two main groups; group 1 included 45 non-dialysis-dependent CKD patients (CKD ND), stages 3–5, and group 2 included 45 patients with CKD on maintenance hemodialysis ≥6 months (CKD 5HD). Each group was subdivided according to the presence or absence of cardiovascular calcification as described in (Table 1).

**Table 1:** Classification of the studied population according to distribution of cardiovascular calcifications.

			**Groups**			**Tests of significance**	
			**G 1 *N* = 45**	**G 2 *N* = 45**	**Total *N* = 90**	**Test statistic**	***P*** **-value**
Cardiovascular calcifications	Yes	*N*	G 1A	G 2A	25	$\chi_{\text{ChS}}^{2}$ = 1.38	0.239
			10	15			
		%	22.2%	33.3%	27.8%		
	No	*N*	G 1B	G 2B	65		
			35	30			
		%	77.8%	66.7%	72.2%		
Vascular calcification^*^			9	13	22 (24.4%)		
Valvular calcification^*^			6	8	14 (15.5%)		

*$\chi_{\text{ChS}}^{2}$, Pearson's Chi square test of association. Significant at P < 0.05*.

* *Some patients have vascular and valvular calcifications*.

**Figure 1:**
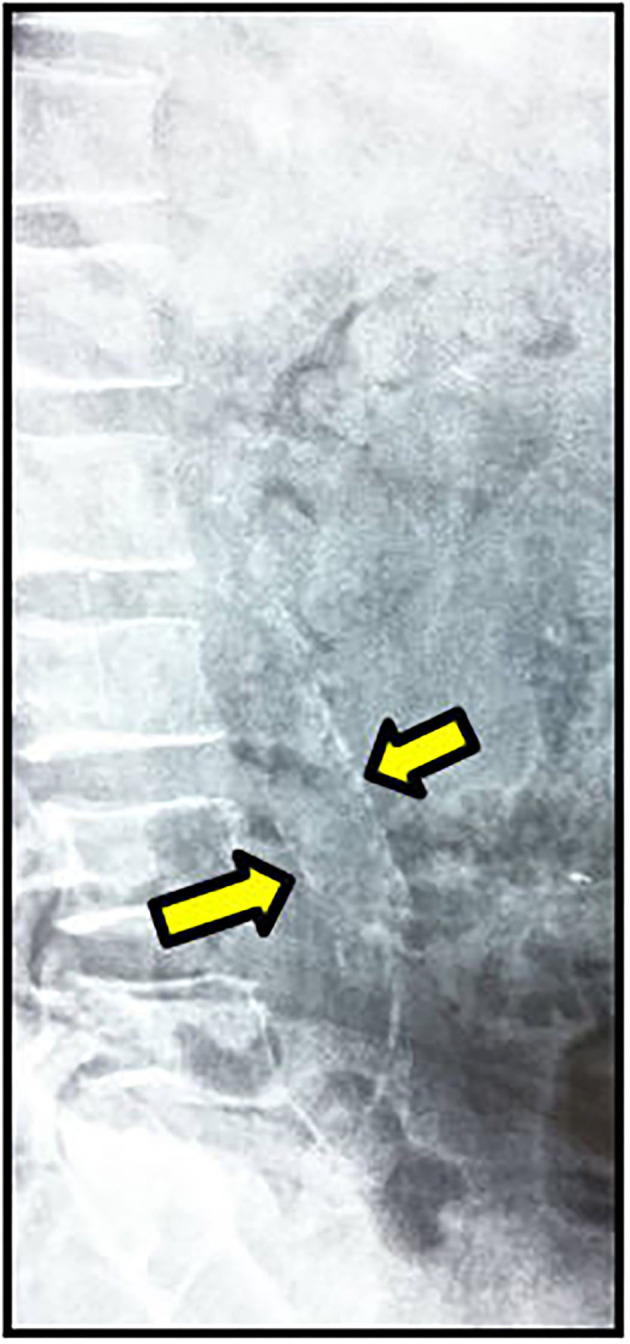
Calcification of anterior and posterior walls of abdominal aorta.

**Figure 2:**
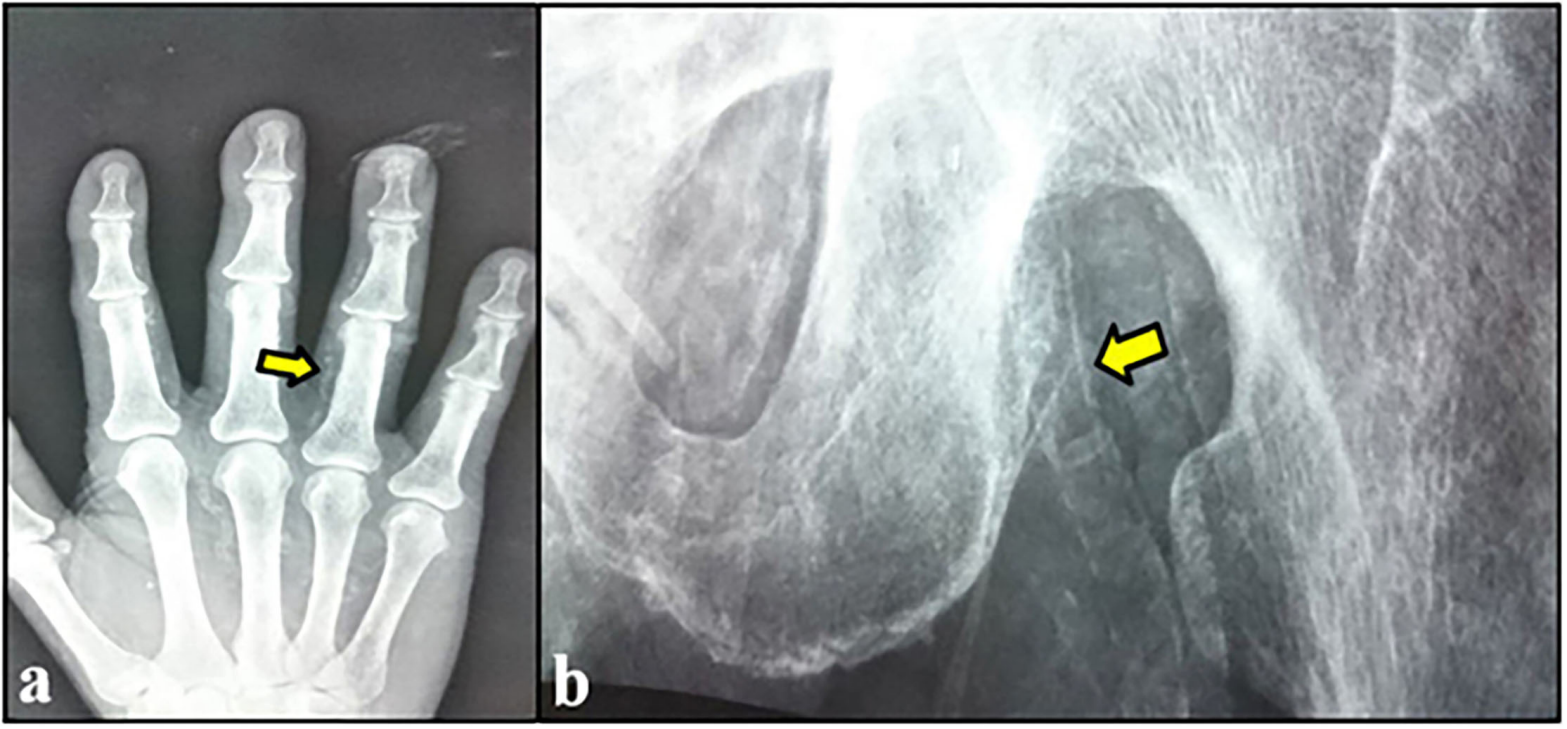
**(A)** Calcification of digital arteries of the hand. **(B)** Left femoral artery calcification.

**Figure 3:**
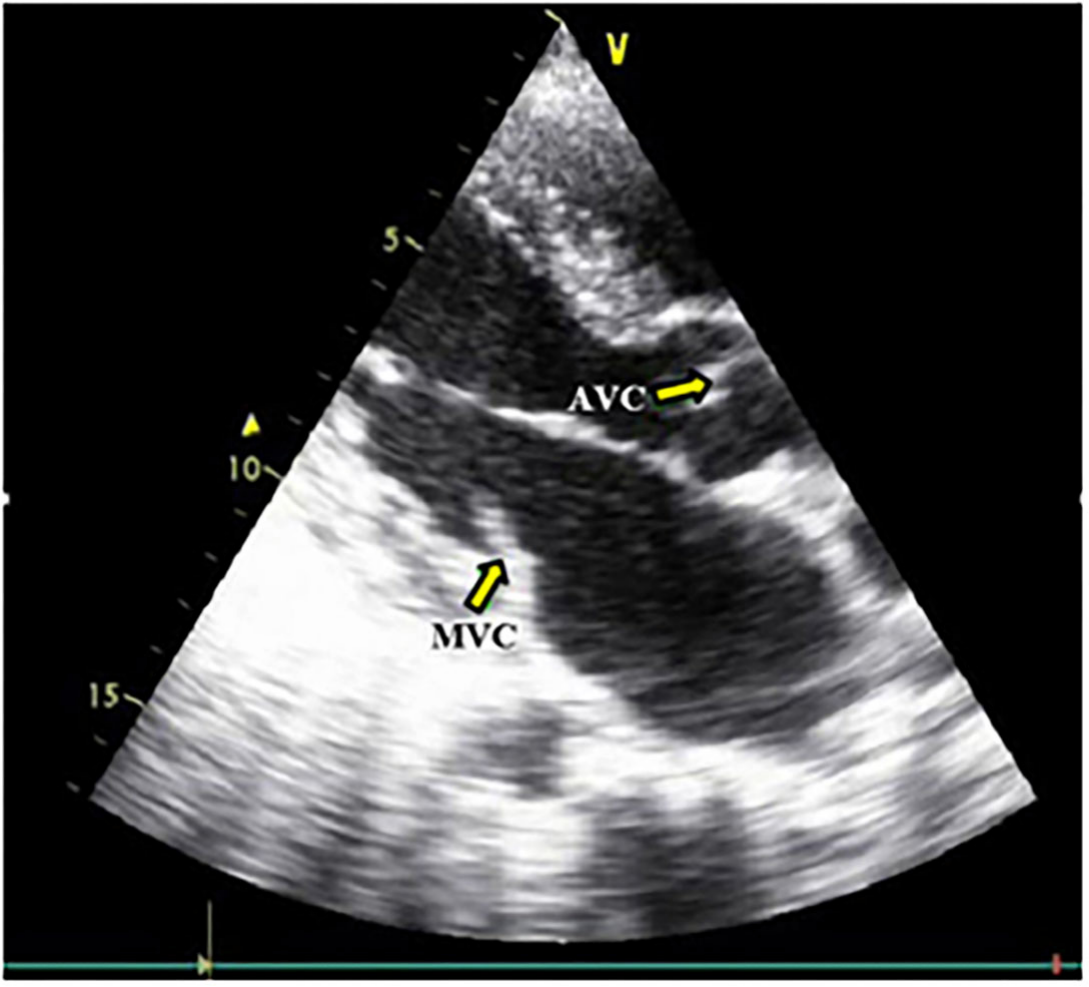
Aortic and Mitral valves calcification. MVC, Mitral valve calcification; AVC, Aortic valve calcification.

According to CKD stages, Patients with cardiovascular calcification increased with the progression of CKD to be highest in stage 5HD. The most common cause of CKD was obstructive uropathy (29%) in group 1 and diabetic kidney disease (26.6%) in group 2. The demographic and clinical characteristics were shown in (Table 2). The extent of CVC according to the affected site was as the following; abdominal aortic calcifications (Figure 1), pelvis and hands calcifications (Figure 2) and valvular calcifications (Figure 3). Comparisons of cardiovascular calcifications sites and scores between group 1A and 2A were included in (Table 3).

**Table 2:** Clinical and demographic characteristics of the studied groups.

			**Groups**				**Tests of significance**	
			**1A *N* = 10**	**1B *N* = 35**	**2A *N* = 15**	**2B *N* = 30**	**Test statistic**	***P*** **-value**
**Age (years)**	Minimum		28	25	32	24	F = 0.074	0.974
	Maximum		60	65	60	65		
	Mean		44.40	46.09	46.20	46.10		
	Standard deviation (SD)		10.78	10.61	9.16	11.97		
**Gender**	M	*N*	4	19	7	14	$\chi_{\text{ChS}}^{2}$ = 0.813	0.846
		%	40.0%	54.3%	46.7%	46.7%		
	F	*N*	6	16	8	16		
		%	60.0%	45.7%	53.3%	53.3%		
**Smoking**	Yes	*N*	2	7	4	4	$\chi_{\text{ChS}}^{2}$ = 1.474	0.697
		%	20.0%	20.0%	26.7%	13.3%		
	No	*N*	8	28	11	26		
		%	80.0%	80.0%	73.3%	86.7%		
**History of CVD**	Yes	*N*	4	8	6	10	$\chi_{\text{ChS}}^{2}$ = 2.318	0.524
		%	40.0%	22.9%	40.0%	33.3%		
	No	*N*	6	27	9	20		
		%	60.0%	77.1%	60.0%	66.7%		
**CKD stages**	Stage 3	*N*	1	11	0	0	$\chi_{\text{ChS}}^{2}$ = 28.754	0.001
		%	10.0%	31.43%	0.0%	0.0%		
	Stage 4	*N*	3	13	0	0		
		%	30.0%	37.14%	0.0%	0.0%		
	Stage 5 ± HD	*N*	6	11	15	30		
		%	60.0%	31.43%	100.0%	100.0%		
**Duration of hemodialysis (years)**	Minimum				1	1	*t* = 0.692	0.493
	Maximum				10.25	16		
	Mean				5.63	6.43		
	Standard deviation (SD)				2.91	3.92		

*F, One way ANOVA test; $\chi_{\text{ChS}}^{2}$, Pearson's Chi square test of association; t, independent samples T-test. Significant at P < 0.05*.

**Table 3:** Comparison of the cardiovascular calcifications sites and scores between group 1A and 2A.

		**Groups**				**Tests of significance**	
**Calcification site**			**1A** ***N*** = **10**		**2A *N* = 15**	**Test statistic**	***P*** **-value**
Abdominal aortic calcification score	*N*	8 (80%)		10 (66.6%)	*t* = 0.363	0.721
	Min—Max		3–12		3–12		
	Mean		7.75		7.20		
	SD		3.41		3.01		
Pelvis and hands calcification score	*N*		5 (50%)		8 (53%)	*t* = 0.11	0.91
	Min—Max		1–3		1–3		
	Mean		2.20		2.25		
	SD		0.84		0.71		
Valvular (aortic & mitral) calcification	Single valve	*N*	2	4	$\chi_{\text{ChS}}^{2}$ = 0.006	0.93
		%	33.3	50		
	Double valves	*N*	4	4		
		%	66.7	50		

*$\chi_{\text{ChS}}^{2}$, Pearson's Chi square test of association; t, independent samples T-test. Significant at P < 0.05*.

Comparisons of the serum laboratory parameters in the studied groups are displayed in (Table 4). Regarding minerals and bone profile, serum total ALP and serum phosphorus were non-significantly higher and serum magnesium was non-significantly lower in calcification groups (1A & 2A) when compared with non-calcification groups (1B & 2B), respectively.

**Table 4:** Comparison of serum laboratory parameters in the studied groups.

		**Groups**				**Tests of significance**	
		**1A *N* = 10**	**1B *N* = 35**	**2A *N* = 15**	**2B *N* = 30**	**Test statistic**	***P*** **-value**
iPTH (pg/ml)	Median	129.5	202	226	196	Z_kw_ = 3.03	0.387
	IQR	95–200	91–368	156–342	96.8–321		
ALP (IU/dl)	Mean	113.30	90.06	109.80	97.43	F = 1.004	0.395
	SD	35.13	24.24	33.84	33.04		
Ca (mg/dl)	Mean	8.13	8.41	8.20	8.32	F = 0.346	0.792
	SD	0.80	0.83	1.06	0.93		
*P* (mg/dl)	Mean	5.64	4.48	5.02	4.47	F Welch = 2.03	0.132
	SD	1.56	1.40	1.57	0.91		
Mg (mg/dl)	Mean	1.75	1.91	1.77	1.88	F = 0.999	0.398
	SD	0.41	0.32	0.35	0.33		
CRP (mg/L)	Median	3	3	6	6	Z_kw_ = 38.44	<0.001
	IQR	3–6	3–4	4–24	4–24		
Total cholesterol (mg/dl)	Median	184.5	137	132	163	Z_kw_ = 2.99	0.393
	IQR	140–230	98–209	92–215	117–220		
Triglycerides (mg/dl)	Median	115	110	124	114	Z_kw_ = 1.47	0.688
	IQR	77–167	82–157	94–180	84–168		
hs-cTnT (ng/L)	Median	216	68.8	74.5	38.25	Z_kw_ = 25	<0.001
	IQR	170–467	32.3–121	59.4–227	23.2–72.6		
OPG (ng/L)	Median	6.85	0.51	7.64	0.60	Z_kw_ = 39.24	<0.001
	IQR	5.98–7.43	0.26–0.89	4.87–7.80	0.26–2.53		

*F, One way ANOVA test; Z${}_{\text{KW}}^{,}$ Kruskall-wallis test; F Welch, Welch ANOVA test. Significant at P <0.05. iPTH, intact parathyroid hormone; ALP, alkaline phosphatase; Ca, calcium; P, phosphorus; Mg, magnesium; CRP, C-reactive protein; hs-cTnT, high sensitivity cardiac troponin T; OPG, osteoprotegerin*.

C-reactive protein was significantly higher in the dialysis groups; groups 2A and 2B when compared with the non-dialysis groups; groups 1A and 1B, respectively. On the other hand, there was no significant difference on comparing calcification vs. non-calcification groups; 1A vs. 1B and 2A vs. 2B. There were no significant differences as regard serum total cholesterol and the median of serum triglycerides among the studied groups.

High sensitivity cardiac troponin T level and serum OPG level in group 1A were significantly higher than that of group 1B. In addition, hs-cTnT and OPG in group 2A were significantly higher than that of group 2B. However, there were no significant differences on comparing groups 1A and 1B (non-dialysis groups) with groups 2A and 2B (dialysis groups), respectively.

**Figure 4:**
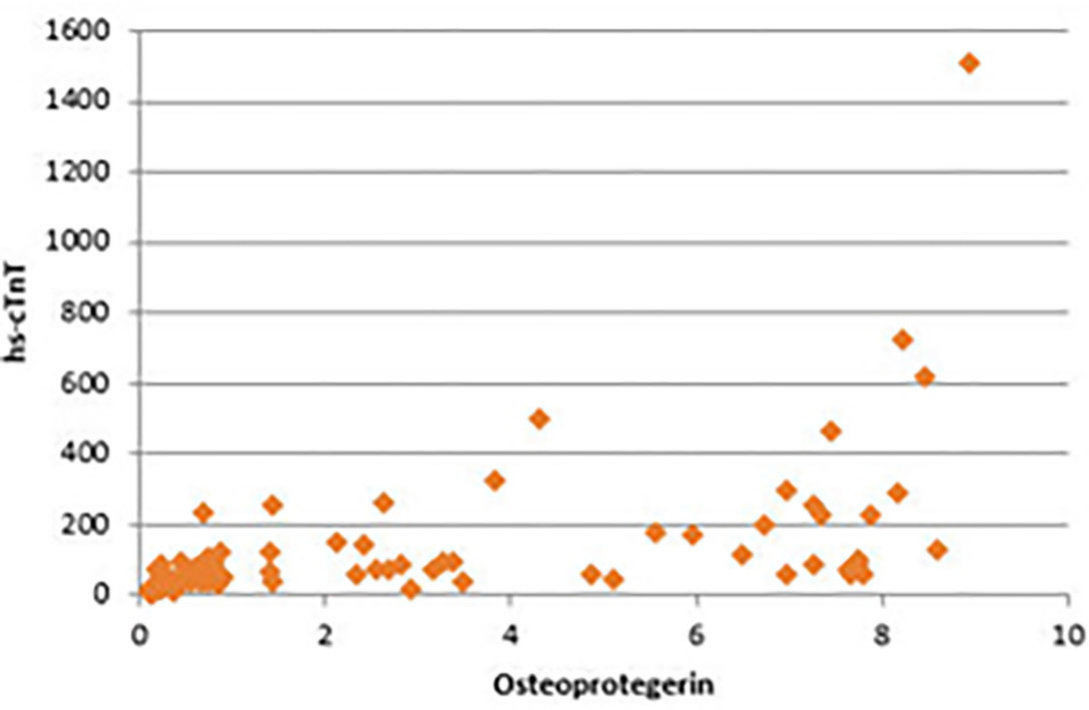
Correlation between serum osteoprotegerin and hs-cTnT.

There were significant positive correlations between serum OPG and serum creatinine, blood urea, CRP, and serum phosphorus. In addition, the strongest positive correlation was present between serum OPG and hs-cTnT (rs = 0.72, *P* < 0.001) (Figure 4). Also, there were significant positive correlations between serum OPG and AACS (*P* < 0.001), pelvis and hands calcification score (*P* < 0.001) and valvular calcification (*P* = 0.032).

There were significant positive correlations between serum hs-cTnT and AACS (*P* < 0.001) and valvular calcification (*P* = 0.032). On the other hand, there was non-significant positive correlation with pelvis and hands calcification score (*P* = 0.279). Significant positive correlations were present between serum phosphorus and AACS (*P* = 0.006) and pelvis and hands calcification score (*P* = 0.032), and non-significant positive correlation with valvular calcification (*P* = 0.162) was noticed. The significant positive correlation of AACS score with serum OPG (rs = 0.879), serum hs-cTnT (rs = 0.772), and phosphorus (rs = 0.616) was the strongest among the all sites affected by calcification.

There were no significant correlations between arterial calcification scores and extent of valvular calcification with other studied laboratory parameters.

There were non-significant negative correlations between serum OPG and age, eGFR, hemoglobin, serum albumin, corrected serum total calcium, and serum magnesium. In addition, there were non-significant positive correlations between serum OPG and duration of dialysis, Kt/V, 24 h urine protein measurement, iPTH, total ALP, total cholesterol and triglycerides.

Binary logistic regression analysis was performed to predict the occurrence of CVC from the studied variables that showed significant association with it. Among the studied variables, serum phosphorus and OPG were found to contribute significantly to the model. The logistic regression model was statistically significant (χ^2^ = 75.13, *P* < 0.001). The model explained 81.7% of the variance and correctly classified 94.4% of cases. Sensitivity was 84%, and specificity was 98.5%. Increased levels of phosphorus and OPG were associated with an increased likelihood of developing CVC (odds ratios: 0.096 and 6.445, respectively).

**Figure 5:**
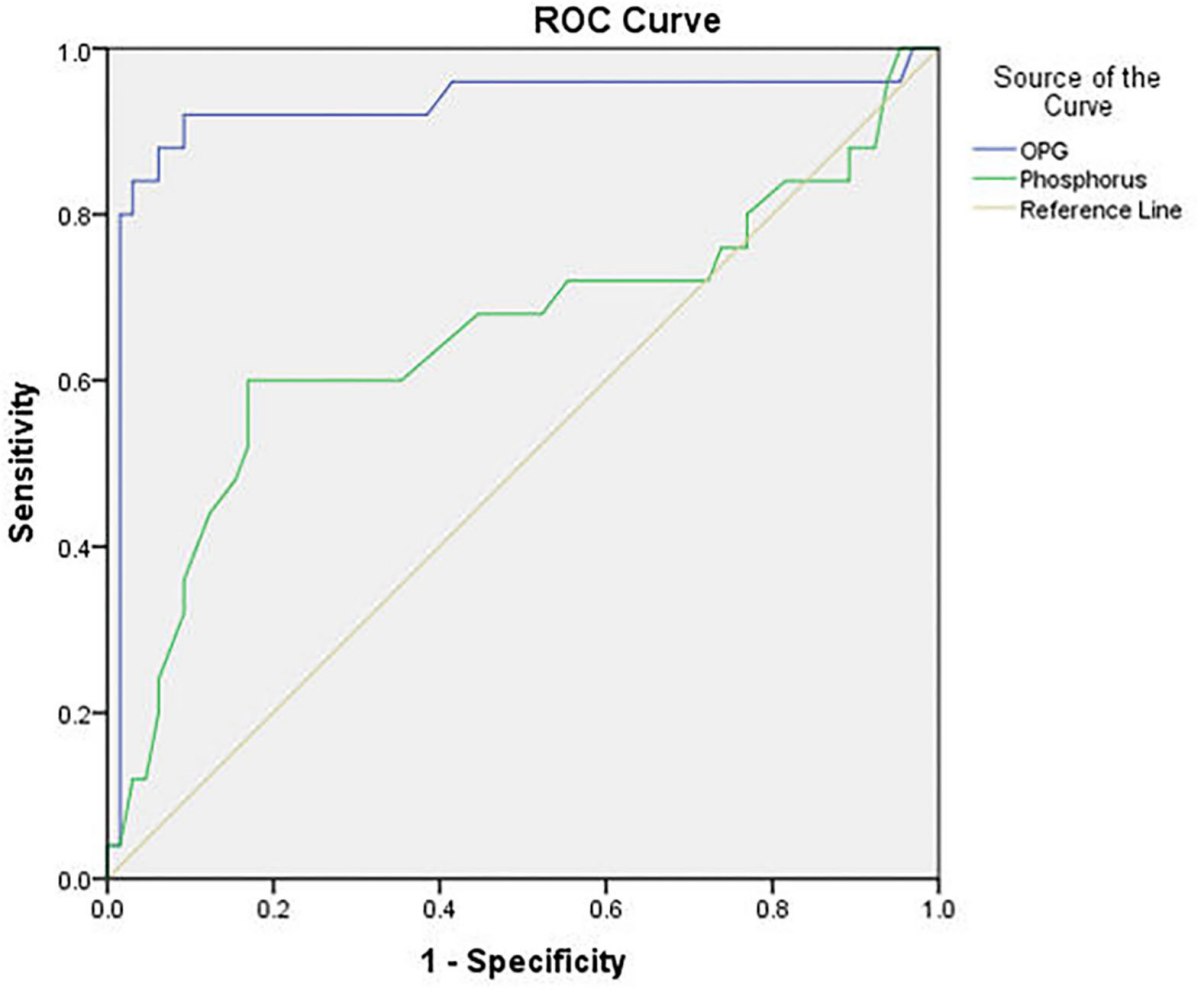
ROC curve of serum osteoprotegerin and phosphorus level in predicting cardiovascular calcification.

At a cut-off value ≥4.6 ng/L, OPG was significantly valid in predicting CVC with sensitivity 84%, specificity 96.9% and overall accuracy 93.3%. At a cut-off value ≥6.95 mg/dl, phosphorus was significantly valid in predicting CVC with sensitivity 12%, specificity 96.9%, and overall accuracy 73.3%. Analysis of receiver operating characteristics (ROC) curves of OPG and phosphorus as predictors of CVC showed an area under the curve (AUC) = 0.926 for OPG (*P* < 0.001), and AUC = 0.654 for phosphorus (*P* = 0.024) (Figure 5).

A multiple regression was done to predict OPG level from serum creatinine, blood urea, CRP, serum hs-cTnT and serum phosphorus. This regression model is a good fit of the data and statistically significantly predicted OPG level (F = 11.58, *P* < 0.001). This model accounted for 35.3% of the variation in OPG level (*R*^2^). But, only serum phosphorus and serum creatinine were independent predictors of OPG and added statistically significant value to the prediction (*P* < 0.001 and = 0.048, respectively). Regression equation is OPG = −2.829 + (0.322 X creatinine) + (1.167 X phosphorus).

## Discussion

In an attempt to tackle this deadly alliance of CKD, mineral and bone disease and CVD, our study was done to evaluate the relationship between the cardiovascular calcification and a myocardial injury biomarker, hs-cTnT, as representatives of cardiovascular burden in CKD and serum osteoprotegerin, an osteoclastogenesis inhibitory factor, which is involved in bone metabolism regulation as well as vascular disease, inflammation and apoptosis.

A system for quantification of vascular calcification (VC) was described by Kauppila et al. (26) in a subgroup of participants of the Framingham heart study. It relies on lateral lumbar radiographs and the calculation of the AACS. Bellasi et al. (27) found that AACS correlated well with electron beam CT scores of coronary arteries in chronic HD patients. Adragao et al. set up a simple vascular calcification score based on plain radiographic films of pelvis and hands. Vascular calcifiction score ≥3 was independently associated with coronary artery disease (*P* = 0.008) and peripheral arterial disease (*P* < 0.001). They concluded that this scoring represents a simple tool for the assessment of cardiovascular risk related to VC in chronic hemodialysis patients (25).

In the current study, the mean AACS was 7.75 ± 3.41 in group 1A and 7.2 ± 3.01 in group 2A, and mean pelvis and hands score was 2.2 ± 0.84 in group 1A and 2.25 ± 0.71 in group 2A. The number of patients with valvular calcification was 6 (two single and four double valvular) in group 1A, and 8 (four single and four double valvular) in group 2A. There were no significant differences regarding the calcification degree in all sites between CKD ND and CKD 5HD patients.

Several publications have reported differences in the prevalence of CVC in CKD patients depending on the world region analyzed, ethnicity and the diagnostic method used. A French paper published by London et al. (28) including 202 patients on chronic hemodialysis reported 68% prevalence of vascular calcifications diagnosed by X-ray and echocardiogram. Toussaint el al. (29) showed 90% prevalence of abdominal aortic calcification in patients on hemodialysis in Australia. Nitta et al. (30) noted 70% prevalence of VC in ESRD patients diagnosed by electron bean CT.

In agreement with our study, Rosa-Diez et al. studied the prevalence of vascular and valvular calcifications in eight dialysis centers in Argentina. They have shown that a higher rate of VC with the use of plain X-ray radiographs (63%) when compared to the prevalence of valvular calcifications (28%) obtained with echocardiogram (31).

Moreover, Honkanen et al. studied the prevalence of abdominal aortic calcification in 933 HD patients obtained from 47 centers in six European countries. Lateral lumbar radiography of the abdominal aorta was used to determine the overall AACS. The mean AACS of the study population was 10.3 ± 0.3 (24).

Similar to our study, Górriz et al. assessed VC using the Kauppila and Adragao scores in 742 non-dialysis-CKD patients. They found that VC is common in patients with CKD (mean eGFR 27 ± 12 ml/min per 1.73 m^2^), with a prevalence of 79% and prominent in 47% (Adragao score ≥3 or Kauppila score >6) (32). Thus in agreement with our results, this study importantly shows that VC starts earlier in the course of CKD, which suggests that it may be potentially modifiable long before the initiation of dialysis. In addition, there was non-significant correlation between eGFR and CVC degree in our study. On the contrary, Budoff et al. found a strong and graded relationship between lower eGFR and increasing coronary artery calcification score, independent of traditional risk factors. This dissimilarity may be due to the different method used for measurement of VC in a different site in the latter study (33).

In the same context, distribution of patients with CVC according to CKD stages in our study revealed that its presence increased with the progression of CKD (decrease in eGFR) to be highest in stage 5HD. This finding was in concordance with the results of Morena et al. who studied the prevalence of coronary artery calcification in CKD patients. 54% of patients had VC, among which 9.7% were at CKD stages 1+2, 38.9% at stage 3 and 51.4% at stages 4+5. The decline in eGFR was significantly associated with an increase in presence VC (34).

In agreement with our findings, Kraus et al. (35) observed that radiographic aortic calcifications did not correlate significantly with dialysis duration, and prevalence of moderate/severe calcification at the aortic valve and mitral valve did not differ significantly by dialysis duration. Additionally, Jean et al. and El Baz et al. (36, 37) reported that Kt/V values were non-significantly different in VC patients from those without VC, and not associated with the VC degree. In contrast to our results, Rosa-Diez et al. (31) noted that dialysis therapy >36 months was significant for Adragao calcification score univariate model. Also, Toussaint et al. and El Baz et al. (29, 37) reported that the presence and severity of abdominal aortic artery calcification were related to the duration of dialysis. This contradiction with our finding can be explained by the lower number of patients with CVC and the non-significant difference of mean dialysis duration between both groups in our study (mean dialysis duration for group IIA = 5.6 and group IIB = 6.4 years).

In our study CRP was significantly higher in dialysis when compared with non-dialysis groups, but there was no significant difference on comparing calcification with non-calcification groups. Caliskan et al. and Turkmen et al. (38, 39) agreed with our result and found no correlation between high sensitive CRP (hs-CRP) levels and VC. Besides, Toussaint et al. (29) reported that there was no significant relationship between AACS and CRP. On the contrary, Ishimura et al. (40) found that CRP was significantly associated with the presence of VC in both aorta and hand arteries. Also, El Baz et al. (37) found significantly high CRP among patients with VC.

Hyperphosphatemia is considered a key determinant of cardiovascular mortality and calcifications progression in CKD patients (41). In concordance with our results, Tomiyama et al. (42) reported that higher levels of serum phosphorus were associated with the presence of severe coronary artery calcification (CAC; *P* = 0.013). Also, El Baz et al. (37) found that patients with VC had higher serum phosphorus than those without VC, and AACS had positive correlation with serum phosphorus. On the contrary, Kazuhiro et al. and Rosa-Diez et al. found that serum phosphorus was not associated with VC (31, 43). Additionally, Russo et al. (44) demonstrated that serum concentrations of phosphorus didn't predict the appearance or progression of CAC. This contrariety can be due to different baseline levels of serum phosphorus and different sites studied for CVC in the different studies.

Russo et al. (44) agreed with our results and demonstrated that serum concentrations of calcium and iPTH didn't predict the appearance or progression of CAC. In addition, Toussaint et al. (45) reported that there were no significant associations between VC and serum markers of mineral metabolism; including calcium and iPTH, and serum lipids. Moreover, Rosa-Diez et al. (31) found no associations between AACS and the levels of serum calcium and iPTH. On the contrary, Moldovan et al. (46) found that serum calcium, and PTH were significantly correlated with VC.

Troponin levels have been shown to increase in a non-linear manner as renal function deteriorates; making the interpretation of elevated troponin levels in patients with CKD and suspected myocardial injury more difficult (47). As regard application of high sensitivity cardiac troponins in the assessment of myocardial injury in CKD population, Twerenbold et al. compared the diagnostic performance of the 0/1-h ESC algorithms of rapid rule-in and rule-out in patients with and without renal impairment using both hs-TnT and hs-TnI. The authors noted that the specificity was significantly lower (hs-TnT: 88.7% vs. 96.5%; hs-TnI: 84.4% vs. 91.7%) with sensitivity remaining relatively unchanged (hs-TnT: 100.0% vs. 99.2%; hs-TnI: 98.6% vs. 98.5%) in patients with and without renal dysfunction, respectively (48).

In concordance with our findings, Jung et al. quantified the level of coronary artery calcium by multirow spiral CT to obtain a CAC score, and reported that severe CAC was detected in 45% of patient. cTnT was independently positively associated with the degree of CAC severity (49). Furthermore, Kitagawa et al. (50) demonstrated that serum hs-cTnT concentration was associated with increased degree of CAC and obstructive CAD, suggesting that serum hs-cTnT may therefore be a marker to detect subclinical atherosclerosis. The average Agatston score was higher in patients with serum hs-cTnT concentration ≥14 ng/L than those with serum hs-cTnT concentration of <14 ng/L (*P* = 0.002).

The elucidation of the OPG/RANKL/RANK system has vastly increased our understanding of the mechanisms underlying the bone remodeling process. This system plays a central role in the pathophysiological mechanisms underlying bone remodeling disorders and CVD (51).

Recently, Marques et al. evaluated the predictive role of OPG for all-cause and cardiovascular mortality in patients with CKD stages 3–5 over a 5-year follow-up period. The cutoff value for OPG determined using ROC was 10 pmol/L for general causes mortality and 10.08 pmol/L for CV causes mortality. Patients with higher serum OPG levels presented with higher mortality rates compared to patients with lower levels, and in multivariate analysis, OPG was a marker of general and cardiovascular mortality independent of sex, age, CVD, diabetes, and CRP levels (52).

In agreement with our results, Nitta et al. found that serum OPG level was significantly greater in patients with higher aortic calcification index (ACI) than in those with lower ACI. There was a positive association between OPG and ACI (*P* < 0.0001). Multiple regression analyses indicated that serum OPG levels were independently associated with the severity of aortic calcification (*P* < 0.0001) (21). Additionally, Moe et al. (53) noted that there was a positive association of coronary artery calcification score with OPG levels (*P* = 0.045).

Furthermore, Mikami et al. reported that CAC score was positively correlated with OPG in a group of diabetic pre-dialysis CKD patients. The ROC curve analysis for coronary artery calcification score (CACS) >200 revealed that the sensitivity and specificity of OPG were 79.4 and 66.7%, respectively, when the cut-off value was set at 1.207 ng/ml (54). In addition, Morena et al. demonstrated that the presence of CAC was significantly associated with high OPG levels. The cut-off value best predicting CAC score was 757.7 pg/ml (sensitivity = 91.7%; specificity = 59.0%) (34). In a following study, Morena et al. (55) noted that a high level of CAC was associated a high level of OPG, and the risk for calcifications was significantly increased when OPG ≥6.26 pmol/L.

In addition, El Baz et al. (37) reported that circulating OPG was significantly higher among hemodialysis patients with VC and was correlated positively with abdominal aortic and coronary artery calcification scores, and Avila et al. (56) concluded that OPG was the strongest risk factor associated with new development and rapid progression of arterial calcification in incident PD patients.

Interestingly, as regards the effect of renal transplantation on VC and OPG, Bargnoux et al. prospectively assessed the evolution of coronary artery calcification and OPG levels after renal transplantation. They found that renal transplantation was accompanied by mineral metabolism improvement with a decrease of OPG mean from 955 to 527 pg/ml. They also reported that baseline OPG level was significantly associated with baseline CAC, but not with CAC progression after 1 year of renal transplantation (57).

Morena et al. agreed with our result and noted that high OPG levels were associated with hs-CRP. While, they disagreed by reporting that increased age, decreased eGFR, calcium, iPTH, and ALP were significantly associated with OPG, while no significant association was found with phosphorus (55). Moreover, El Baz et al. (37) agreed with our results and demonstrated positive correlations between OPG and serum phosphorus and CRP.

In contradiction with our findings, they noted that OPG correlated positively with the duration of hemodialysis, ALP and PTH. But, Chonchol et al. (58) contradicted our result by finding a positive weak correlation between OPG and both total cholesterol and triglycerides. In addition, Azar et al. (59) reported a significant relationship of OPG with age and calcium level while PTH was not shown to be significant.

In agreement with our results, Ford et al. reported a significant association between higher plasma OPG levels and hs-cTnT and hs-CRP. OPG was independently associated with hs-cTnT in a cohort of asymptomatic patients with CKD, suggesting a role for a known mediator of bone and mineral metabolism in the pathogenesis of myocardial dysfunction (60). Also, Nascimento et al. (61) noted that OPG was positively associated with cardiac troponin I, and concluded that elevated levels of serum OPG might be associated with atherosclerosis and all-cause mortality in patients with CKD.

Moreover, Shetelig et al. studied temporal profile of OPG in 272 symptomatic STEMI patients treated with primary percutaneous coronary intervention (PCI). Cardiac MRI was performed in the acute phase and after 4 months. They concluded that OPG was found to be associated with myocardial injury (62).

In the same context, Sigrist et al. investigated a total of 134 subjects [60 hemodialysis, 28 peritoneal dialysis (PD) and 46 CKD stage 4]. VC was measured using multi-slice spiral CT of the superficial femoral artery. They reported that OPG >25 pmol/L was significant predictor of mortality (63). Additionally, Omland et al. (64) studied the prognostic value of serum OPG in patients with ACS, and concluded that OPG is strongly predictive of long-term mortality and heart failure development in patients with ACS, independent of conventional risk markers. This was supported by Scialla et al. (17) who stated that higher OPG was associated with higher mortality over up to 13 years of follow-up of 602 chronic HD patients.

Noteworthy, higher levels of OPG have been reported in patients with vascular damage, suggesting that an increase in OPG level may represent a compensatory self-defensive mechanism against factors that promote VC, atherosclerosis and other forms of vascular damage (65).

Limitations of the current study include that the association among OPG, cardiovascular calcification and hs-cTnT in the present study does not suggest the causal relationship, due to its cross-sectional design. Study design as single baseline laboratory values may lessen the strength of some of the significant predictors of cardiovascular calcification especially those that vary widely day-to-day. Treatment effect on cardiovascular calcification was not assessed. In addition, the number of included patients is quite low.

## Conclusions

Chronic kidney disease is associated with fatal cardiovascular consequences in part due to ectopic calcification of soft tissues particularly arteries and cardiac valves. In addition to, occurrence of silent myocardial injury that can be diagnosed by elevated hs-cTnT level on serial follow up. A positive association of OPG with CVC and hs-cTnT, and a positive association between CVC and hs-cTnT were found in the current study.

This suggests a role for a known mediator of mineral and bone metabolism (OPG) in the pathogenesis and risk stratification of cardiovascular calcification and myocardial injury with a potential role as a therapeutic target. The bone-vascular cross talk might open the possibility to develop new methods for preventive and therapeutic interventions to reduce both bone loss and cardiovascular adverse outcomes.

## Data Availability Statement

The raw data supporting the conclusions of this article will be made available by the authors, without undue reservation.

## Ethics Statement

The studies involving human participants were reviewed and approved by the Research Ethics Committee of Faculty of Medicine Tanta University. The patients/participants provided their written informed consent to participate in this study.

## Author Contributions

KO, MHA, WE, and SA: manuscript preparation, editing, review, and literature research. RM, SM, and NS: data acquisition and analysis. MAE and MHE: clinical studies and literature research. MA, AA, ME, and OS: echocardiography. HM, DA, and NB: laboratory work. SSAM and SAE: data analysis and statistical analysis. All authors contributed to the concept and design of the study and all approved the final version of the manuscript.

## Conflict of Interest

The authors declare that the research was conducted in the absence of any commercial or financial relationships that could be construed as a potential conflict of interest.

## Publisher's Note

All claims expressed in this article are solely those of the authors and do not necessarily represent those of their affiliated organizations, or those of the publisher, the editors and the reviewers. Any product that may be evaluated in this article, or claim that may be made by its manufacturer, is not guaranteed or endorsed by the publisher.

## Abbreviations

AACS: abdominal aortic calcification score
ACI: aortic calcification index
CAC: coronary artery calcification
CKD: chronic kidney disease
CKD 5HD: chronic kidney disease on maintenance hemodialysis
CKD ND: non-dialysis-dependent chronic kidney disease patients
CVC: cardiovascular calcification
CVD: cardiovascular disease
ESKD: End-stage kidney disease
hs-cTnT: high sensitivity cardiac troponin T
OPG: osteoprotegerin
RANKL: receptor activator of nuclear factor-κB ligand
VC: vascular calcification.

## References

[1] Di Lullo L Barbera V Bellasi A Cozzolino M De Pascalis A Russo D. Vascular and valvular calcifications in chronic kidney disease: an update. EMJ Nephrol. (2016) 4:84–91. Available online at: https://www.emjreviews.com35656113

[2] Tonelli M Wiebe N Culleton B House A Rabbat C Fok M. Chronic kidney disease and mortality risk: a systematic review. J Am Soc Nephrol. (2006) 17:2034–47. 10.1681/ASN.2005101085 16738019

[3] Berl T Henrich W. Kidney-heart interactions: epidemiology, pathogenesis, and treatment. Clin J Am Soc Nephrol. (2006) 1:8–18. 10.2215/CJN.00730805 17699186

[4] Go AS Chertow GM Fan D McCulloch CE Hsu C-y. Chronic kidney disease and the risks of death, cardiovascular events, and hospitalization. N Engl J Med. (2004) 351:1296–305. 10.1056/NEJMoa041031 15385656

[5] Weiner DE Tighiouart H Elsayed EF Griffith JL Salem DN Levey AS. The Framingham predictive instrument in chronic kidney disease. J Am Coll Cardiol. (2007) 50:217–24. 10.1016/j.jacc.2007.03.037 17631213

[6] Van Der Zee S Baber U Elmariah S Winston J Fuster V. Cardiovascular risk factors in patients with chronic kidney disease. Nat Rev Cardiol. (2009) 6:580–9. 10.1038/nrcardio.2009.121 19621012

[7] Jankowski J Floege J Fliser D Böhm M Marx N. Cardiovascular disease in chronic kidney disease: pathophysiological insights and therapeutic options. Circulation. (2021) 143:1157–72. 10.1161/CIRCULATIONAHA.120.050686 33720773 PMC7969169

[8] Kidney Disease: Improving Global Outcomes (KDIGO) CKD-MBD Work Group. KDIGO clinical practice guideline for the diagnosis, evaluation, prevention, and treatment of chronic kidney disease-mineral and bone disorder (CKD-MBD). Kidney Int Suppl. (2009) 113:S1–130. 10.1038/ki.2009.188 19644521

[9] Goodman WG London G Amann K Block GA Giachelli C Hruska KA. Vascular calcification in chronic kidney disease. Am J Kidney Dis. (2004) 43:572–9. 10.1053/j.ajkd.2003.12.005 14981617

[10] Mizobuchi M Towler D Slatopolsky E. Vascular calcification: the killer of patients with chronic kidney disease. J Am Soc Nephrol. (2009) 20:1453–64. 10.1681/ASN.2008070692 19478096

[11] Antman E Bassand JP Klein W Ohman M Sendon JL Rydén L. Myocardial infarction redefined—a consensus document of the joint European Society of Cardiology/American College of Cardiology committee for the redefinition of myocardial infarction. J Am Coll Cardiol. (2000) 36:959–69. 10.1016/S0735-1097(00)00804-4 10987628

[12] Dubin RF Li Y He J Jaar BG Kallem R Lash JP. Predictors of high sensitivity cardiac troponin T in chronic kidney disease patients: a cross-sectional study in the chronic renal insufficiency cohort (CRIC). BMC Nephrol. (2013) 14:229. 10.1186/1471-2369-14-229 24148285 PMC4016297

[13] Aakre KM Røraas T Petersen PH Svarstad E Sellevoll H Skadberg Ø. Weekly and 90-min biological variations in cardiac troponin T and cardiac troponin I in hemodialysis patients and healthy controls. Clin Chem. (2014) 60:838–47. 10.1373/clinchem.2013.216978 24619542

[14] Kang E Ryu H Kim J Lee J Lee KB Chae DW. Association between high-sensitivity cardiac troponin T and echocardiographic parameters in chronic kidney disease: results from the KNOW-CKD cohort study. J Am Heart Assoc. (2019) 8:e013357. 10.1161/JAHA.119.013357 31514574 PMC6818004

[15] Simonet W Lacey D Dunstan C Kelley M Chang M-S Lüthy R. Osteoprotegerin: a novel secreted protein involved in the regulation of bone density. Cell. (1997) 89:309–19. 10.1016/S0092-8674(00)80209-3 9108485

[16] Boyle WJ Simonet WS Lacey DL. Osteoclast differentiation and activation. Nature. (2003) 423:337–42. 10.1038/nature01658 12748652

[17] Scialla JJ Kao WL Crainiceanu C Sozio SM Oberai PC Shafi T. Biomarkers of vascular calcification and mortality in patients with ESRD. Clin J Am Soc Nephrol. (2014) 9:745–55. 10.2215/CJN.05450513 24458076 PMC3974354

[18] Cozzolino M Ureña-Torres P Vervloet MG Brandenburg V Bover J Goldsmith D. Is chronic kidney disease-mineral bone disorder (CKD-MBD) really a syndrome? Nephrol Dial Transplant. (2014) 29:1815–20. 10.1093/ndt/gft514 24516228

[19] Levey AS Bosch JP Lewis JB Greene T Rogers N Roth D. A more accurate method to estimate glomerular filtration rate from serum creatinine: a new prediction equation. Ann Intern Med. (1999) 130:461–70. 10.7326/0003-4819-130-6-199903160-00002 10075613

[20] Daugirdas JT. Second generation logarithmic estimates of single-pool variable volume Kt/V: an analysis of error. J Am Soc Nephrol. (1993) 4:1205–13. 10.1681/ASN.V451205 8305648

[21] Nitta K Akiba T Uchida K Otsubo S Takei T Yumura W. Serum osteoprotegerin levels and the extent of vascular calcification in haemodialysis patients. Nephrol Dial Transplant. (2004) 19:1886–9. 10.1093/ndt/gfh263 15128884

[22] Apple FS Collinson PO IFCC Task Force on Clinical Applications of Cardiac Biomarkers. Analytical characteristics of high-sensitivity cardiac troponin assays. Clin Chem. (2012) 58:54–61. 10.1373/clinchem.2011.165795 21965555

[23] Wilson PW Kauppila LI O'donnell CJ Kiel DP Hannan M Polak JM. Abdominal aortic calcific deposits are an important predictor of vascular morbidity and mortality. Circulation. (2001) 103:1529–34. 10.1161/01.CIR.103.11.1529 11257080

[24] Honkanen E Kauppila L Wikström B Rensma PL Krzesinski J-M Aasarod K. Abdominal aortic calcification in dialysis patients: results of the CORD study. Nephrol Dial Transplant. (2008) 23:4009–15. 10.1093/ndt/gfn403 18676346 PMC2639067

[25] Adragao T Pires A Lucas C Birne R Magalhaes L Gonçalves M. A simple vascular calcification score predicts cardiovascular risk in haemodialysis patients. Nephrol Dial Transplant. (2004) 19:1480–8. 10.1093/ndt/gfh217 15034154

[26] Kauppila LI Polak JF Cupples LA Hannan MT Kiel DP Wilson PW. New indices to classify location, severity and progression of calcific lesions in the abdominal aorta: a 25-year follow-up study. Atherosclerosis. (1997) 132:245–50. 10.1016/S0021-9150(97)00106-8 9242971

[27] Bellasi A Ferramosca E Muntner P Ratti C Wildman R Block G. Correlation of simple imaging tests and coronary artery calcium measured by computed tomography in hemodialysis patients. Kidney Int. (2006) 70:1623–8. 10.1038/sj.ki.5001820 16955104

[28] London GM Guerin AP Marchais SJ Métivier F Pannier B Adda H. Arterial media calcification in end-stage renal disease: impact on all-cause and cardiovascular mortality. Nephrol Dial Transplant. (2003) 18:1731–40. 10.1093/ndt/gfg414 12937218

[29] Toussaint ND Pedagogos E Lau KK Heinze S Becker GJ Beavis J. Lateral lumbar X-ray assessment of abdominal aortic calcification in Australian haemodialysis patients. Nephrology. (2011) 16:389–95. 10.1111/j.1440-1797.2010.01420.x 21054667

[30] Nitta K Ogawa T. Vascular calcification in end-stage renal disease patients. In: Nitta K editor. Chronic Kidney Diseases-Recent Advances in Clinical and Basic Research. Vol. 185. Basel: Karger Publishers (2015). p. 156–67. 10.1159/000380980

[31] Rosa-Diez G Bratti G Filannino G Peñalba A Otreras F Ledesma M. Prevalence of factors related to vascular calcification in patients with chronic kidney disease on dialysis. Medicina. (2017) 77:207–13. Available online at: http://www.scielo.org.ar 28643678

[32] Górriz JL Molina P Cerverón MJ Vila R Bover J Nieto J. Vascular calcification in patients with non-dialysis CKD over 3 years. Clin J Am Soc Nephrol. (2015) 10:654–66. 10.2215/CJN.07450714 25770175 PMC4386255

[33] Budoff MJ Rader DJ Reilly MP Mohler ER Lash J Yang W. Relationship of estimated GFR and coronary artery calcification in the CRIC (Chronic Renal Insufficiency Cohort) study. Am J Kidney Dis. (2011) 58:519–26. 10.1053/j.ajkd.2011.04.024 21783289 PMC3183168

[34] Morena M Dupuy A-M Jaussent I Vernhet H Gahide G Klouche K. A cut-off value of plasma osteoprotegerin level may predict the presence of coronary artery calcifications in chronic kidney disease patients. Nephrol Dial Transplant. (2009) 24:3389–97. 10.1093/ndt/gfp301 19574342

[35] Kraus MA Kalra PA Hunter J Menoyo J Stankus N. The prevalence of vascular calcification in patients with end-stage renal disease on hemodialysis: a cross-sectional observational study. Ther Adv Chronic Dis. (2015) 6:84–96. 10.1177/2040622315578654 25984289 PMC4416967

[36] Jean G Bresson E Terrat J-C Vanel T Hurot J-M Lorriaux C. Peripheral vascular calcification in long-haemodialysis patients: associated factors and survival consequences. Nephrol Dial Transplant. (2008) 24:948–55. 10.1093/ndt/gfn571 18852190

[37] El Baz TZ Khamis OA El-Shehaby A Chahine H Ahmed AA Alsawasany MA. Relationship between serum osteoprotegerin and vascular calcifications in hemodialysis patients. Egypt Heart J. (2017) 69:149–55. 10.1016/j.ehj.2017.02.004 29622969 PMC5839346

[38] Caliskan Y Demirturk M Ozkok A Yelken B Sakaci T Oflaz H. Coronary artery calcification and coronary flow velocity in haemodialysis patients. Nephrol Dial Transplant. (2010) 25:2685–90. 10.1093/ndt/gfq113 20190240

[39] Turkmen K Gorgulu N Uysal M Ozkok A Sakaci T Unsal A. Fetuin-A inflammation, and coronary artery calcification in hemodialysis patients. Indian J Nephrol. (2011) 21:90. 10.4103/0971-4065.82128 21769170 PMC3132345

[40] Ishimura E Okuno S Kitatani K Maekawa K Izumotani T Yamakawa T. C-reactive protein is a significant predictor of vascular calcification of both aorta and hand arteries. Semin Nephrol. (2004) 24:408–12. 10.1016/j.semnephrol.2004.06.008 15490400

[41] Ishimura E Okuno S Taniwaki H Kizu A Tsuchida T Shioi A. Different risk factors for vascular calcification in end-stage renal disease between diabetics and non-diabetics: the respective importance of glycemic and phosphate control. Kidney Blood Press Res. (2008) 31:10–15. 10.1159/000112542 18097148

[42] Tomiyama C Higa A Dalboni MA Cendoroglo M Draibe SA Cuppari L. The impact of traditional and non-traditional risk factors on coronary calcification in pre-dialysis patients. Nephrol Dial Transplant. (2006) 21:2464–71. 10.1093/ndt/gfl291 16735378

[43] Yamada K Fujimoto S Nishiura R Komatsu H Tatsumoto M Sato Y. Risk factors of the progression of abdominal aortic calcification in patients on chronic haemodialysis. Nephrol Dial Transplant. (2007) 22:2032–7. 10.1093/ndt/gfm031 17395663

[44] Russo D Palmiero G De Blasio AP Balletta MM Andreucci VE. Coronary artery calcification in patients with CRF not undergoing dialysis. Am J Kidney Dis. (2004) 44:1024–30. 10.1053/j.ajkd.2004.07.022 15558523

[45] Toussaint ND Lau KK Strauss BJ Polkinghorne KR Kerr PG. Associations between vascular calcification, arterial stiffness and bone mineral density in chronic kidney disease. Nephrol Dial Transplant. (2007) 23:586–93. 10.1093/ndt/gfn031 17933842

[46] Moldovan D Moldovan I Rusu C Racasan S Patiu IM Brumboiu A. Vascular calcifications and renal osteodystrophy in chronic hemodialysis patients: what is the relationship between them? Int Urol Nephrol. (2011) 43:1179–86. 10.1007/s11255-010-9841-5 20862543

[47] Chesnaye NC Szummer K Bárány P Heimbürger O Magin H Almquist T. Association between renal function and troponin T over time in stable chronic kidney disease patients. J Am Heart Assoc. (2019) 8:e013091. 10.1161/JAHA.119.013091 31662068 PMC6898818

[48] Twerenbold R Badertscher P Boeddinghaus J Nestelberger T Wildi K Puelacher C. 0/1-h triage algorithm for myocardial infarction in patients with renal dysfunction. Circulation. (2018) 137:436–51. 10.1161/CIRCULATIONAHA.117.028901 29101287 PMC5794234

[49] Jung HH Ma KR Han H. Elevated concentrations of cardiac troponins are associated with severe coronary artery calcification in asymptomatic haemodialysis patients. Nephrol Dial Transplant. (2004) 19:3117–23. 10.1093/ndt/gfh488 15546893

[50] Kitagawa N Okada H Tanaka M Hashimoto Y Kimura T Tomiyasu K. High-sensitivity cardiac troponin T is associated with coronary artery calcification. J Cardiovasc Comput Tomogr. (2015) 9:209–14. 10.1177/2047487316632364 25843242

[51] Vega D Maalouf NM Sakhaee K. The role of receptor activator of nuclear factor-κB (RANK)/RANK ligand/osteoprotegerin: clinical implications. J Clin Endocrinol Metab. (2007) 92:4514–21. 10.1210/jc.2007-0646 17895323

[52] Marques GL Hayashi S Bjällmark A Larsson M Riella M Olandoski M. Osteoprotegerin is a marker of cardiovascular mortality in patients with chronic kidney disease stages 3–5. Sci Rep. (2021) 11:1–9. 10.1038/s41598-021-82072-z 33510348 PMC7844415

[53] Moe SM Reslerova M Ketteler M O'Neill K Duan D Koczman J. Role of calcification inhibitors in the pathogenesis of vascular calcification in chronic kidney disease (CKD). Kidney Int. (2005) 67:2295–304. 10.1111/j.1523-1755.2005.00333.x 15882271

[54] Mikami S Hamano T Fujii N Nagasawa Y Isaka Y Moriyama T. Serum osteoprotegerin as a screening tool for coronary artery calcification score in diabetic pre-dialysis patients. Hypertens Res. (2008) 31:1163–70. 10.1291/hypres.31.1163 18716364

[55] Morena M Jaussent I Dupuy A-M Bargnoux A-S Kuster N Chenine L. Osteoprotegerin and sclerostin in chronic kidney disease prior to References 201 dialysis: potential partners in vascular calcifications. Nephrol Dial Transplant. (2015) 30:1345–56. 10.1093/ndt/gfv081 25854266

[56] Avila M Mora C del Carmen Prado M Zavala M Paniagua R. Osteoprotegerin is the strongest predictor for progression of arterial calcification in peritoneal dialysis patients. Am J Nephrol. (2017) 46:39–46. 10.1159/000477380 28614819

[57] Bargnoux AS Dupuy AM Garrigue V Jaussent I Gahide G Badiou S. Evolution of coronary artery calcifications following kidney transplantation: relationship with osteoprotegerin levels. Am J Transplant. (2009) 9:2571–9. 10.1111/j.1600-6143.2009.02814.x 19775319

[58] Kendrick J Chonchol M. The role of phosphorus in the development and progression of vascular calcification. Am J Kidney Dis. (2011) 58:826–34. 10.1053/j.ajkd.2011.07.020 21956015 PMC3199354

[59] Azar SA Nakhjavani MRJ Ethemadi J Malekian M Haghjo AG. Correlation of serum osteoprotegerin levels and bone mineral density early after parathyroidectomy in hemodialysis patients. Int J Curr Res Aca Rev. (2014) 2:196–201. Available online at: http://www.ijcrar.com

[60] Ford ML Smith ER Tomlinson LA Chatterjee PK Rajkumar C Holt SG. FGF-23 and osteoprotegerin are independently associated with myocardial damage in chronic kidney disease stages 3 and 4. Another link between chronic kidney disease–mineral bone disorder and the heart. Nephrol Dial Transplant. (2011) 27:727–33. 10.1093/ndt/gfr316 21750158

[61] Nascimento M Hayashi SY Riella M Lindholm B. Elevated levels of plasma osteoprotegerin are associated with all-cause mortality risk and atherosclerosis in patients with stages 3–5 chronic kidney disease. Braz J Med Biol Res. (2014) 47:995–1002. 10.1590/1414-431X20144007 25296363 PMC4230291

[62] Shetelig C Limalanathan S Eritsland J Hoffmann P Seljeflot I Gran JM. Osteoprotegerin levels in ST-elevation myocardial infarction: temporal profile and association with myocardial injury and left ventricular function. PloS ONE. (2017) 12:e0173034. 10.1371/journal.pone.0173034 28253327 PMC5333871

[63] Sigrist MK Levin A Er L McIntyre CW. Elevated osteoprotegerin is associated with all-cause mortality in CKD stage 4 and 5 patients in addition to vascular calcification. Nephrol Dial Transplant. (2009) 24:3157–62. 10.1093/ndt/gfp253 19491380

[64] Omland T Ueland T Jansson AM Persson A Karlsson T Smith C. Circulating osteoprotegerin levels and long-term prognosis in patients with acute coronary syndromes. J Am Coll Cardiol. (2008) 51:627–33. 10.1016/j.jacc.2007.09.058 18261681

[65] Kiechl S Schett G Wenning G Redlich K Oberhollenzer M Mayr A. Osteoprotegerin is a risk factor for progressive atherosclerosis and cardiovascular disease. Circulation. (2004) 109:2175–80. 10.1161/01.CIR.0000127957.43874.BB 15117849

